# Research on the Evolutionary Game of Construction and Demolition Waste (CDW) Recycling Units’ Green Behavior, Considering Remanufacturing Capability

**DOI:** 10.3390/ijerph18179268

**Published:** 2021-09-02

**Authors:** Xingwei Li, Ruonan Huang, Jiachi Dai, Jingru Li, Qiong Shen

**Affiliations:** College of Architecture and Urban-Rural Planning, Sichuan Agricultural University, Du Jiangyan, Chengdu 611830, China; 201808468@stu.sicau.edu.cn (R.H.); 2020325016@stu.sicau.edu.cn (J.D.); jingruli2020@outlook.com (J.L.)

**Keywords:** CDW recycling supply chain, remanufacturing, evolutionary game, corporate leadership

## Abstract

At present, China has not yet formed an effective development model for the industrialization of construction waste. The level of construction waste treatment and resource utilization is still low, and recycled products also lack market competitiveness. In order to promote the effective development of the remanufactured construction and demolition waste supply chain better, and based on the present situation, this manuscript establishes a game model for recycling units in two different situations: with and without remanufacturing capabilities. However, most existing studies have determined that all recycling units have remanufacturing capabilities. In the first situation, the main players of the game are recycling units with remanufacturing capabilities and consumers. In the second situation, the main players of the game are recycling units without remanufacturing capabilities and the third-party remanufacturer with remanufacturing ability. Therefore, our research can ascertain the optimal strategy choices of both parties in the game under different return situations and discuss the impact of changes to related parameters through numerical simulations. The results show: (1) When the recycling unit has remanufacturing capabilities, corporate leadership and government supervision rate have positive effects on its evolution to strict manufacturing. Only a high supervision rate can effectively suppress the negative impact of speculative gains and drive the system to (strict manufacturing; positive). Furthermore, the higher the supervision rate, the faster the system will converge. Then, the consumer’s payment difference coefficient has a negative effect on the evolution of the recycling unit to strict manufacturing. The larger the payment difference coefficient, the faster the system will converge to a stable state (tendency to formal manufacturing; negative). (2) When the recycling unit does not have the ability to remanufacture, the government cost subsidy rate and the recycling unit’s effort profit coefficient have positive effects on the recycling unit’s evolution to the direction of effort. Meanwhile, the larger the profit coefficient of the recovery unit’s effort, the faster the system will converge. The conclusions obtained provide certain theoretical guidance for the decision making of CDW recycling supply chain recovery units and relevant government departments.

## 1. Introduction

At present, China is in a critical period, beginning its “14th Five-Year Plan” and advancing the construction of ecological civilization, achieving high-quality development, and making good progress towards carbon peak and carbon neutrality. Effectively improving the level of comprehensive utilization of bulk solid waste is an important measure to achieve the goals of the “14th Five-Year Plan” for national economic and social development [1]. At present, the remanufacturing industry tends to be mature in foreign countries. At the same time, with the active introduction of various domestic policies, the prospects for progress in China are good.

Green development takes efficiency, coordination, and sustainability as its goals, with the government, enterprises, and residents as the main body [2]; it is a new manifestation of sustainable development [3]. As a social system including the economy and the natural environment [4], green behavior mainly emphasizes the systematization of both [5]. Faced with the environmental problems of rapid economic and social development, the necessity of green development has become a broad consensus in global development [6]. By improving the level of green technology, enterprises can adapt to the general trend of green development [7]. Green behavior consists of environmentally sustainable behavior [8] and is an important part of green development research, involving all aspects of production and life [9]. The specific performance can be measured by clean production behavior, green supply chain management practice, energy-saving behavior, green consumption, household waste disposal, and recycling of products [10,11].

Compared with other remanufacturing industries, the construction and demolition waste (CDW) remanufacturing industry is still in its infancy and still faces many problems in development, construction, and operation. Existing research shows that due to the lack of supporting regulations and industry policies, many fledgling companies are operating in a difficult situation [12]; for example, return on investment and accumulation of enterprise development cannot be guaranteed, the supply of raw materials for production is unstable, and furthermore, consumers have concerns about the inherent quality of products and environmental protection issues, etc. These difficulties require the government to provide actual support. In addition, due to the low benefit of recycled building products and the meager corporate profits, many companies are unwilling to manufacture recycled CDW products. As a result, it is difficult for a closed-loop recycling and remanufacturing supply chain to achieve high-quality development. In China, the current recovery rate of CDW is 5%, but in developed countries such as Japan and the nations of the EU, the recovery rate of CDW has exceeded 90% [13]. Therefore, in order to alleviate these problems, China has done a lot of work in supporting the development of garbage circulation. Since 2016, national policies involving the recycling of construction waste have been frequently introduced, pointing to future directions for the development of the construction waste treatment industry. The “Several Opinions on Further Strengthening the Management of Urban Planning and Construction” for the first time explicitly put forward a timetable for the basic establishment of a construction waste recycling and recycling system within around five years. Cities across China have also responded to the national call; for instance, the Xi’an Municipal Government provided compensation and support for industries related to construction waste treatment through government funding, tax exemption, or other tax incentives, loan guarantees [14], etc.; Shenzhen and other cities not only encouraged the use of recycled construction waste products but also listed recycled products in the green product catalog and government green procurement catalog. To a certain extent, these measures have supported the development of enterprises that recycle energy-saving building materials.

The decision making of supply chain stakeholders is the key to promoting the better formation and operation of China’s closed-loop CDW recycling supply chain. At the same time, the government plays an important role in supply chain management. By adjusting supervision probability and penalties, the government can control the quality of recycled CDW products. Through the adjustment of subsidized funds for consumers, the government has an impact on the enthusiasm of consumers’ participation, thus affecting the decision-making behaviors of CDW recycling supply chain stakeholders. Therefore, the government’s role should be considered during the decision-making process of the closed-loop supply chain. Many scholars have studied game behavior between stakeholders of the CDW recycling supply chain. Yang et al. [15] studied the problem of recycled products’ deceptive prices in closed-loop supply chains and constructed a variety of evolutionary game models composed of vendors and consumers. Based on the government’s rewards and punishment, Long et al. [16] constructed an evolutionary game model between production units and recycling units and then discussed the evolutionary stability strategies under different scenarios. Both of the aforementioned studies showed that if the government actively inspects reproduction markets, while increasing the rewards, punishments, and government subsidies, then the government can effectively promote the development of the CDW reproduction market. However, there is not much literature considering the impact of corporate leadership and consumer green preferences on the CDW recycling supply chain. Current studies have shown that leadership has a positive impact in terms of improving performance [17], so the impact of corporate leadership on enterprises’ decisions in the CDW recycling supply chain should be considered. Similarly, consumers’ green preferences have a significant impact on their green purchasing behavior [18], so the impact of consumer green preferences is also essential in the CDW recycling supply chain.

**Figure 1:**
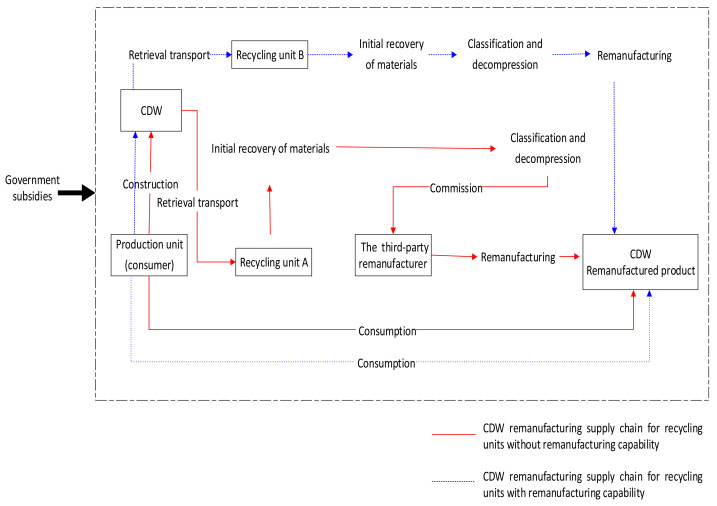
Flow chart of remanufactured CDW products’ supply chain.

The CDW recycling industry chain is a closed-loop supply chain. Figure 1 shows the business process, which begins with construction units that produce the construction waste during the construction process. Meanwhile, as the supply chain needs to use related materials to complete some construction work, it may have another identity as the consumer of remanufactured CDW products. In the game process, the consumer refers to the construction unit. Then, there are usually two ways for CDW waste to become reusable construction products: First, it can be converted into reusable products after undergoing processes such as recycling and sorting, compressing, cleaning, and testing implemented by the recycling unit. The other way is that when the recycling unit does not have the ability to remanufacture a type of CDW waste, it can perform some simple tasks such as collecting and sorting the waste. Then, the third-party remanufacturer (3PR) with remanufacturing ability can purchase the materials handled by the recycling unit and make them into new products. Once reaching the national standard, it can be sold to the construction unit again. After the remanufactured products are used by the construction unit, waste is generated again; thus, a new round of remanufacturing activities begins.

Based on the current situation, this manuscript considers two game situations if the recycling unit has remanufacturing capabilities: (1) When the recycling unit has remanufacturing capabilities, it will play with consumers. Because of the lack of standards in industry quality and remanufacturing technology, companies can produce non-standard products; for example, the strength may not be up to standard or hazardous materials may not be cleaned up. Companies may also uphold a high degree of social responsibility and produce remanufactured CDW products of high quality. Therefore, there are two strategies that the recycling unit may employ (strictly producing remanufactured CDW products or the tendency to formally produce remanufactured CDW products). At the same time, consumers have differences in their attitudes to remanufactured products. There are consumers who strongly support and actively buy remanufactured products, while there are also consumers who are “forced” to buy them due to external factors. Thus, consumers have two strategies (actively participating in the CDW recycling supply chain or passively participating in the CDW recycling supply chain). This will affect the development of the CDW recycling industry supply chain in the collection and use of raw materials and sales channels. Therefore, this manuscript establishes a game model between recycling units and consumers. (2) When the recycling unit does not have remanufacturing capabilities, the recycling unit cannot produce remanufactured CDW products. However, in order to obtain further profits, the recycling unit will then choose to cooperate with a 3PR. According to the China Building Materials Network [19], construction waste recycling companies with disposal capacity often face the dilemma of having no source of materials due to lack of construction waste raw materials; in this case, 3PRs are willing to cooperate with recycling units. Since the recycling unit is not a direct beneficiary of the CDW recycling industry supply chain at this time, it may not actively classify and dismantle construction waste. As a result, it is difficult for 3PRs to maintain consistent quality of their processed products, resulting in a vicious cycle in the construction waste recycling industry. Therefore, this manuscript assumes that both the recycling unit and the 3PR unit have strategic choices (high-quality effort; low-quality effort). The recycling unit, based on its quality efforts, can ensure that the raw materials that reach the 3PR unit are not mixed materials and do not need to be sorted again by the 3PR; the 3PR unit, based on its own quality efforts, can ensure that the product quality is up to standard.

Thus, for recycling units with remanufacturing capabilities, is it better to strictly manufacture or, to maximize their own interests, to choose formal manufacturing? For recycling units that do not have remanufacturing capabilities, is it better to commit to high-quality efforts or to only consider their own interests by choosing low-quality efforts? For 3PRs who are direct stakeholders, is it better to commit to high-quality efforts or to choose low-quality efforts and not consider whether the product reaches expected standards? For consumers, is it better to actively support and spontaneously use remanufactured products or to only purchase remanufactured CDW products in special circumstances? These have become urgent problems that must be solved quickly in the CDW recycling supply chain.

This manuscript introduces corporate leadership into the evolutionary game model for the first time, studying the optimal decision-making process of the CDW recycling supply chain in the two situations: (1) recycling units and consumers, and (2) recycling units and 3PRs. On the one hand, our study enriches the literature on evolutionary games and government supervision, providing a theoretical basis for the decision-making processes of CDW recycling units in other countries or regions. On the other hand, our study is based on the remanufacturing capability of CDW recycling units, providing a decision-making basis for implementing optimal policies in different situations, which is not available from existing research. In addition, it provides certain theoretical guidance to help governments formulate relevant policies, such as supervision methods and subsidies, that can be adopted to better promote recycling units to strictly produce remanufactured products and encourage consumers’ active participation while incentivizing recycling units and 3PRs to choose high-quality efforts.

The rest of this manuscript is as follows: Section 2 reviews the relevant literature. Section 3 establishes the evolutionary game models of recycling units with remanufacturing capacity and consumers and of recycling units without remanufacturing capacity and 3PRs. Section 4 analyzes the stability of each equilibrium point, determines the evolutionary stability strategy (ESS) in different situations, and discusses the influence of related parameters on the evolutionary path via numerical simulation. Section 5 summarizes the relevant conclusions and limitations of this manuscript.

## 2. Literature Review

### 2.1. Closed-Loop Supply Chain for Recycling and Remanufacturing

China attaches great importance to the development of the remanufacturing industry and clearly proposes to “standardize the development of the remanufacturing industry” in the “14th Five-Year Plan”, thus incorporating the remanufacturing industry into strategic emerging industries. At present, a large number of studies focus on closed-loop supply chains for recycling and remanufacturing, mainly involving the choice of recycling channels, inventory issues, and strategic issues such as recycling, quality, price, etc., in order to achieve cost minimization and satisfy remanufacturing needs [20,21,22].

Reproduction is the process of implementing the high-tech repair and transformation of waste products. Generally, remanufactured products are not inferior to the original products in terms of quality and performance [23]. Most early studies assumed that there was no difference in quality between the remanufactured product and the original product. However, over time, the quality, quantity, and price issues involved in recycling quickly attracted the attention of researchers. Guide et al. [20] introduced the concept of product recycling management, qualified how to manage the uncertainty of the quality of recycling, and conducted an economic value analysis. Based on the uncertainty of a remanufactured product’s quality, Fleischmann et al. [24] established a simulation model to assess different reverse logistics structures. Savaskan et al. [25] established a closed-loop supply chain consisting of a manufacturer and a retailer under the assumption that consumers do not perceive any difference between remanufactured and non-remanufactured products and also considered the contrast between retailer recycling and third-party recycling; finally, it was found that retailer recycling is more efficient than third-party recycling in the closed-loop supply chain. Based on market segmentation, Wang et al. [26] further discussed the decision issues faced by closed-loop supply chains in both retailer recycling and third-party recycling of waste products. Vorasayan and Ryan [27] studied the relationship between the optimal pricing and recovery quantity of the remanufactured product and discussed the impact of changes in recycling quality and cost on the optimal pricing strategy. In general, due to the important role of closed-loop supply chains in improving resource cycle utilization rates, reducing waste emissions, and reducing environmental pollution [25], studies on closed-loop supply chains have received general attention from both management circles and academic circles, at home and abroad, and a series of profound research results have been obtained, although further supplements are still needed. 

### 2.2. Green Behavior and Recycling of Construction Waste as a Response to Government Rewards and Punishment

Construction and demolition waste is usually regarded as a treatable economic problem [28]. CDW is the general term for engineering muck, engineering mud, engineering refuse, demolition refuse, and decoration refuse [29]. According to the present situation of CDW treatment, it can be divided into three approaches: reuse, waste landfill, and recycling [30]. The recycling approach is for recyclable waste. In China, the key factors for CDW recovery include specific laws and regulations, environmental awareness, and the supply of CDW recovery units [31]. Green behavior can be expressed through recycling behavior, which is also an important part of green development behavior [10]. Therefore, recycling behavior can influence green behavior.

According to the summary of the previous literature, the behavior of CDW recycling is distinct from the recycling behavior of other waste products, and the difference is mainly reflected in three aspects: First, the objects are different. For example, unlike waste paper recycling, which can be applied to everyday waste, CDW recycling behavior can only be applied to the construction industry. China produces more than 2.5 billion tons of CDW annually [32]. Improper management of a large quantity of CDW will cause harm to society, the environment, and economic resources [33]. Second, the parties involved in the recycling behavior are different. The stakeholders of CDW recycling activities are the government, waste production units (demolition contractors and construction contractors), recycling units, and third-party manufacturing units [32]; these constitute a closed-loop supply chain of CDW recycling behavior. Finally, the effect of recycling is also different; CDW recycling can effectively save resources, reduce pollution, stimulate the economy, and achieve greater social, economic, and environmental effects [34,35]. In a word, the effects of the recycling of construction and demolition waste are distinctive.

Through research, it can be seen that the current construction waste treatment methods in China mainly consist of simple landfills, in addition to incineration, recycling treatment, cycling treatment, and other methods. Due to the diversity of construction waste varieties, there are also differences in their resource treatment methods. For example, concrete, brick, and other stone waste products mainly use mobile crushing and screening methods, and waste, recovered products, etc., are often stored and classified on site. According to Guo [36], the cost of building waste resource utilization needs to consider the following processes: on-site classification cost, transportation cost, pretreatment cost, regeneration cost, and residue landfill. Therefore, for the recycling unit, the resource utilization of construction waste is often time-consuming and labor-intensive. At present, the price of natural raw materials in China is relatively low, which results in the cost of remanufactured products being much higher than the cost of virgin materials, and most companies are reluctant to engage in this kind of costly business. Therefore, such remanufacturing projects require the government’s strong support; only if the government introduces relevant compensation and supervision policies will there be a company willing to remanufacture. Existing studies on government reward and punishment policies show that (1) the probability of government subsidy has a clear incentive effect on the recycling unit, while the probability of supervision is significant in increasing the positive participation of production units [27]; (2) government subsidies for recycling companies are not always necessary because the behavior of recycling companies will be affected by construction companies [37]; and (3) the benefits of subsidized units depend on the government’s financial capacity. Once government subsidy incentives are insufficient to offset the high cost of remanufactured products, the recycling unit may play the role of manufacturing in order to increase its income. Therefore, the government can control the quality of remanufactured products via punishment, imposing fines on recycling units whose products fall short of standards at spot checks. However, from a practical point of view, it is not possible for the government to oversee every company, so companies tend to use formal manufacturing in order to improve their income, consequently causing consumers to reduce future purchases. This behavior further affects the development of the CDW recycling utilization supply chain. In summary, establishing a reasonable governmental reward and punishment mechanism will have a positive impact on the CDW remanufacturing supply chain. At the same time, there are cases in which the government is involved in research on remanufacturing, mostly focused on government subsidies for enterprises but less concerned with government subsidies for consumers [38,39,40]. In reality, consumers are the promoters of reproductive reform. Consumers’ recognition and acceptance of remanufactured products are undoubtedly one of the factors that manufacturers need to consider in production [41]. If the consumer has not yet formed consumer awareness, and the acceptance of remanufactured products remains low in the market, then the production of remanufactured goods will be difficult to advance, potentially causing companies to gradually reduce the production of remanufactured goods. In the real remanufactured market, the impact of government implementation of consumption subsidies on consumers’ willingness to buy green products may be more directly significant. Therefore, this manuscript considers the influences of government reward and punishment mechanisms on recycling units, 3PRs, and consumers. 

### 2.3. Consumers’ Green Preferences and Production Unit Leadership Level

In September 2020, the Sixth China International Cooperation Committee on the environment and development proposed the “14th Five-Year Plan”. Through government-led business entities and public actions, this plan aims to establish a green consumption policy system, focusing on providing society with “efficient, environmentally friendly, and low-carbon” green products and services and encouraging consumers to practice green consumption behaviors and cultivate green lifestyles [42]. Hence, our study encourages consumers to adopt the habit of actively buying remanufactured products. Consumers’ recognition and acceptance of remanufactured products are important factors affecting the supply chain of construction waste recycling. Studies have shown that the profits of supply chain companies are positively correlated with consumers’ green preferences [43]; consequently, we can assume that the higher the green preference of consumers, the more willing they will be to pay higher prices for remanufactured CDW products, and vice versa. At the same time, increasing public awareness of green environmental protection can promote the adoption of green marketing models in the supply chain [44]. Therefore, it is necessary to explore the impact of two types of consumers on the supply chain. From the perspective of the production of CDW units, in order to expand their own interests and reduce costs, companies will choose to tend towards formal manufacturing or not to produce remanufactured CDW products at all, which is not conducive to green development. In fact, companies can reduce costs by improving management practices or via technological innovation. Most of the existing research about the method to reduce the costs of remanufacturing aims to (1) improve the design of existing products by adopting innovation in the production process [45], such as by using advanced equipment [46], processes, and materials, or by using value engineering methods [47]; (2) solve the multi-objective optimization problems in the process planning stage by using genetic algorithms or other methods [37]; or (3) develop a proper remanufacturing quality strategy based on consumer preferences [48]. However, few studies focus on the level of leadership. Leadership, as an ability to motivate and guide employees to establish and realize a common vision, is increasingly valued by corporate managers. Studies on leadership and team performance posit that, in learning organizations, shared leadership has played a stronger role in promoting learning than vertical leadership [49]; however, there are also studies that argue that the significance of shared leadership in promoting team performance is significantly less than that of vertical leadership [50]. This manuscript mainly considers the factor of shared leadership level. With different leadership levels, the performance of the company will also be different, which ultimately affects the company’s decision making.

### 2.4. Expectation Inconsistency Theory

The expectation inconsistency theory (also called the performance difference theory) was proposed by Oliver [51], who argued that that before receiving the product, customers will form an expected value judgment on the utility and performance of the product—that is, an “expectation”; after the actual use of or contact with the product, a real evaluation of the purchased product will be made, and it will be compared with previous expectations. If the real perception after purchase is greater than their own expectations, the customer will have a psychological state of “positive difference”, which will bring customer satisfaction; if the real perception after purchase is less than their prior expectations, it will produce a mental state of “negative difference”, which will produce customer dissatisfaction; if the two are exactly equal, there will be an “indifference” mental state, and the customer will show basic satisfaction at this time. In the 1990s, more and more scholars began to study the inconsistency of expectations; thus far, there are two ways to define gaps in expectations in academia: One is the subtraction gap, which is the perceived performance minus the algebraic gap formed by the comparison standard [52]; the other is the subjective gap, which is the customer’s subjective evaluation of the extent to which the perceived performance exceeds the pre-consumption standard [53]. For most consumption processes, it is difficult to accurately measure the gap between perceived performance and comparison standards; therefore, most studies—ours included—use a gap model of inconsistent subjective expectations. 

According to the expectation inconsistency model proposed by Olshavsky, Miller [54], and Anderson [55], we know that customers will estimate the benefit and utility of the product before purchasing, which is the process of forming “expectations”. After receiving the product, consumers will unconsciously compare its real performance level with the expectation previously envisioned, and the discrepancy between the real and the imaginary is called “inconsistency”. This theory is mostly used in studies on consumer satisfaction [55,56], public service [57], and perceptual performance [57]. According to some research, including a study by Zhao et al. [58], the original intention of loyal customers is to obtain more trust benefits, social benefits, special treatment benefits, etc., from this kind of good relationship; after service failures occur, their perception of symbolic loss may become stronger. Therefore, consumers who actively participate will have unsatisfactory mental states when the recycling unit tends towards formal manufacturing, which is a perceived loss for consumers. Furthermore, if consumers who actively participate have a higher evaluation of the product itself, then they may have a mental state of “no difference” for strictly manufactured products. For consumers who passively participate, they do not have enough trust in remanufactured products and do not have strong environmental awareness, so their expected value is not high. After being exposed to products that tend to be manufactured in form, the real perception obtained by the consumer may be the same as their previous expectation, resulting in a mental state of “no difference”; however, after being exposed to strictly remanufactured products, the real perception of purchase is greater than expected, and consumers who passively participate will generate perceived benefits.

## 3. Model Building

### 3.1. Model Assumptions

Under the condition of not changing the essence of the problem, some complicated conditions are simplified, and the following assumptions are made for the model:

Suppose that there are three game subjects involved in the evolutionary game—namely, the recycling unit, the consumer, and the third-party remanufacturer. The three parties are all economic entities with bounded rationality, with the goal of maximizing their own interests.

According to the research of Wen [59], Chen [60], Hai [61], and others, remanufactured products can effectively alleviate idle resources and environmental pollution problems; thus, if the government effectively stimulates the operation of this industry, it will develop different financial subsidy policies for different targets. Consequently, the government will always support the development of the remanufactured CDW production industry. It can be assumed that the government will subsidize units that use construction waste to produce remanufactured products and consumers who purchase remanufactured products. Moreover, in this paper, government subsidies to recycling units come before the sale of the products, and only when consumers purchase the remanufactured CDW products can they receive subsidies.

In the first case, the recycling unit has the ability to remanufacture CDW. At this time, there are two choices of behaviors: strictly producing remanufactured products or a tendency to formally produce remanufactured products; these two strategies are referred to as “strictly manufacture” (AP) and “tendency to formal manufacturing” (F), respectively.

Consumers’ strategic choices include both active and passive participation in the CDW recycling supply chain, referred to as “active” (A) and “negative” (N), respectively. Active means that under the influence of willingness to contribute to environmental protection and green preferences, consumers will actively purchase remanufactured products regardless of whether there are government subsidies or the price of the remanufactured product(s). Negative refers to consumers who do not trust the quality of reproduced products; only when the prices of remanufactured products are low and they enjoy subsidies from national policies will these consumers choose to buy remanufactured products. Existing research has indicated that [62], in a given period of time, consumers with different willingness to buy new products remain stable for a certain time. Thus, it is assumed that the consumer buys the same number of products in the case of active and negative participation. Therefore, no matter what the consumer’s attitude is towards the CDW production unit, the recycling unit will receive the same basic income from selling remanufactured CDW products.

The leadership of the management in the recycling unit is also related to the additional cost of producing remanufactured CDW products. According to different sources, company leadership is mainly divided into vertical leadership and shared leadership [63]. Vertical leadership comes from formal leaders, while shared leadership comes from informal leaders (employees). The two types of leadership may complement one another in the organization; for example, shared leadership plays an important role in the growth and development phase of the team, while vertical leadership plays an important role in the stability phase of the team [64]. It is assumed that the recycling unit in this research is in the growth stage, so our study only considers shared leadership. Shared leadership behavior has a direct effect on performance, and this relationship has been proven [65]. Therefore, the higher the degree of shared leadership and the higher the enthusiasm of employees, the lower the cost of producing remanufactured CDW products, and vice versa. In order to comply with reality, suppose that the actual cost of producing CDW remanufactured products is as follows: $C_{r1,2}=(1\;-{\;b)C}_{R1,2}$, where $C_{R1}\;\mathrm{and}\;C_{R2}$ are the costs required when vertical leadership is equal to 0, which is related to shared leadership; and b is the leadership of the management (0<b<1).

Due to the heterogeneity of consumers’ perception of product quality [66], different consumers have different perceptions of material quality, while the highest price that consumers are willing to pay is also different. Thus, actively participating consumers are willing to pay higher prices for the purchase of remanufactured CDW products, while passively participating consumers are not. Under the condition of information asymmetry, limited by their own knowledge, experience, and ability, passively participating consumers can easily perceive the uncertainty of the quality of remanufactured CDW products in the process of judging their true quality and the performance of producers and sellers. Assuming that the highest price that consumers are willing to pay for actively participating is $I_{C}$, then the larger the$\;I_{C}$, the higher the price that consumers are willing to pay, and the greater their green preference. The highest price that passively participating consumers are willing to pay is as follows: $\delta I_{C}$, where $\delta \in \left(0,1\right)$, and δ reflects the difference in green preference of different consumers. The smaller δ is, the greater the difference between the prices that the two types of consumers are willing to pay for products of the same quality.

According to the discussion of the theory of inconsistency of expectations in Section 2, our study assumes that consumers will experience perceived loss $D_{C}$ when they actively participate and when recycling units tend towards formal manufacturing. Conversely, when consumers are passively involved, and recycling units engage in strict manufacturing, consumers generate perceived benefits $E_{C}$. In order to simplify the model, it is assumed that consumers do not produce perceived gains or losses when they actively participate and recycling units strictly manufacture, or when the consumers passively participate and recycling units tend towards formal manufacturing. Existing studies have shown [67] that the degree of dissatisfaction caused by the customer’s perceived performance level being lower than their expectation is higher than the degree of satisfaction caused by the perceived performance level being higher than the customer’s expectation at the same range. Therefore, the loss $D_{R}$ generated by the recycling unit is greater than the profit $E_{R}$.

In the second situation, since the recycling unit has no remanufacturing capability, a third-party manufacturer with remanufacturing capability must be introduced. The recycling unit charges material fees to the 3PR, and the third-party remanufacturer is responsible for the production and sale of remanufactured CDW products. For better development of the construction waste recycling supply chain, recycling units and 3PRs need to work together. Assume that the recycling unit and the 3PR strategy selection are denoted as (high-quality effort; low-quality effort)—denoted as (E, NE). For recycling units, efforts include actively formulating recycling management and supervision systems to improve the quantity and quality of CDW’s initial recycling within a given time; for 3PRs, efforts are embodied in strictly implementing safe production standards, carrying out technological innovation, shortening remanufacturing time, improving product quality, etc. Therefore, although selecting high-quality effort means increasing costs, at the same time, it will also bring increasing profits. Previous research [67] shows that the higher the consumers’ recognition of the quality of the product, the higher the profit of the remanufacturing activities. For 3PRs, the increased benefits come from intangible sources such as increased consumer trust, purchase willingness, and social reputation. Enterprises will only remanufacture when profits are positive, so $\pi_{pi}>0$.

Because the revenue-sharing evolutionary contract can realize supply chain coordination [68], our study introduces such a contract model. Assume that there is a cooperative incentive system between the recycling unit and the third-party remanufacturer (the government encourages the cooperative system). There is a linear incentive contract between 3PRs and recyclers, as follows [69]: $S\left(\pi_{p}\right)$ = M +$\;\beta \pi_{p}$, where$\;\pi_{p}$ is the profit obtained by the 3PR from using recycled CDW to produce remanufactured products, M is the fixed income of the recycler—that is, the income from the sale of CDW recyclables to the 3PR, which has nothing to do with $\pi_{p}$, and $M_{r}>0$; β is the share of output profit shared by the remanufacturer from the remanufacturing of waste products. The profit of the third-party remanufacturer $\pi_{p}$ is related to the effort level k of the recycling unit (0<k<1). To simplify the model, suppose $\pi_{p}=\left(1+k\right)\pi_{pi},i=1,2$, and that this is in line with general economic assumptions.

Dynamic and flexible ex post rewards can prevent speculative behavior of social capital and restrain its negative behavior; this method can also better show the effects of government incentives [70]. Thus, suppose that the government subsidy is an ex post reward, the subsidy rate is r, the subsidy fee is C∗r, and C is the cost to the 3PR to produce remanufactured products (not including the cost of purchasing primary recycled materials from recycling units). The subsidy is intended to promote enterprises’ production efforts, as well as to promote the further development of the CDW remanufacturing supply chain, rather than to promote enterprises to engage in remanufacturing.

The assumptions mentioned above are summarized in Table 1.

### 3.2. Model Parameters

To study the problem, the following parameters were defined; Table 2 provides definitions of the parameters involved in the assumptions in the first case. Table 3 shows the game profit matrix between recycling units and consumers in the first situation.

(1) $$u_{r1}=I_{R}+S_{R}-\left(1-b\right)C_{R1}$$

(2) $$u_{c1}=I_{C}-C_{C}+S_{C}$$

(3) $$u_{r2}=I_{R}+S_{R}-\left(1-b\right)C_{R}{}_{1}+E_{R}$$

(4) $$u_{c2}=\delta I_{C}-C_{C}+S_{C}+E_{C}$$

(5) $$u_{r3}=I_{R}+S_{R}-\left(1-b\right)C_{R}{}_{2}-aP_{R}-D_{R}$$

(6) $$u_{c3}=I_{C}-C_{C}+S_{C}-D_{C}$$

(7) $$u_{r4}=I_{R}+S_{R}-\left(1-b\right)C_{R}{}_{2}-aP_{R}$$

(8) $$u_{c4}=\delta I_{C}-C_{C}+S_{C}$$

Note that $u_{ri}$ and $u_{cj}$(i=1,2,3,4,j=1,2,3,4) refer to the recycling units’ profit and consumers’ profit, respectively, when they make different choices.

Table 4 provides definitions of the parameters involved in the assumptions in the second case. Table 5 shows the profit matrix between the recycling unit and the 3PR in the second situation.

(9) $$u_{r1}=\left(1+k\right)\beta \pi_{p2}+M-C_{r}-∆C_{r}$$

(10) $$u_{p1}{=(1+k)\pi }_{p2}{(1-\beta )-M+(C}_{p}{+\Delta C}_{p})s\;$$

(11) $$u_{r2}=\left(1+k\right)\beta \pi_{p1}+M-C_{r}-∆C_{r}$$

(12) $$u_{p2}=\left(1+k\right)\pi_{p2}\left(1-\beta \right)-M+C_{p}s$$

(13) $$u_{r3}=\beta \pi_{p2}+M-C_{r}$$

(14) $$u_{p3}=\pi_{p2}+M-C_{R}$$

(15) $$u_{r4}=\beta \pi_{p1}+M-C_{r}$$

(16) $$u_{p4}=\pi_{p1}\left(1-\beta \right)-M+C_{p}s\;$$

Note that $u_{ri}$ and $u_{pj}$(i=1,2,3,4,j=1,2,3,4) refer to the recycling units’ profit and the 3PR’s profit, respectively, when they make different choices.

## 4. Evolutionary Game Model Analysis

### 4.1. The First Situation, the Game between Recycling Units and Consumers

#### 4.1.1. Calculation of the Stable Point

According to Table 1, the expected benefits of the recycling units in the two strategies of AP and F are as follows:

(17) $$U_{AP}=z\left[I_{R}+S_{R}-\left(1-b\right)C_{R1}\right]+\left(1-z\right)\left[I_{R}+S_{R}+E_{R}-\left(1-b\right)C_{R1}\right]$$

(18) $$U_{F}=z\left[I_{R}+S_{R}-\left(1-b\right)C_{R2}-D_{R}\right]+\left(1-z\right)\left[I_{R}+S_{R}-\left(1-b\right)C_{R2}\right]$$

Consumers’ benefits for the two choices of *A* and *N* are as follows:

(19) $$U_{A}=x\left(I_{C}-C_{C}+S_{C}\right)+\left(1-x\right)\left(I_{C}-C_{C}+S_{C}-D_{C}\right)$$

(20) $$U_{N}=x\left(\delta I_{C}-C_{C}+S_{C}+E_{C}\right)+\left(1-x\right)\left(\delta I_{C}-C_{C}+S_{C}\right)$$

Replicator dynamics equations can describe the evolution of game players’ strategies over time. According to the asymmetric replicator dynamics equations proposed by Taylor and Jonker in 1978 [71], the replicator dynamics equation of the CDW recycling unit for the strategy of AP and that of the consumers for the strategy of A are as follows:

(21) $$F\left(x\right)=\frac{dx}{dt}=x\left(1-x\right)\left(U_{AP}-U_{F}\right)=x\left(1-x\right)\left[\left(1-b\right)\left(C_{R2}-C_{R1}\right)+E_{R}+aP_{R}+z\left(D_{R}-E_{R}\right)\right]$$

(22) $$F\left(z\right)=\frac{dz}{dt}=z\left(1-z\right)\left(U_{A}-U_{N}\right)=z\left(1-z\right)[\left(I_{C}\left(1-\delta \right)-D_{C}+x\left(D_{C}-E_{C}\right)\right]$$

According to the stability theory of first-order differential equations, if *dx*/*dt* = 0 and *dz*/*dt* = 0, the stable points of the system composed of Equations (21) and (22) can be obtained, i.e., (0, 0), (0, 1), (1, 0), and (1, 1). Naturally, (x*, z*) = ($\frac{D_{C\;}-I_{C}\left(1-\delta \right)}{D_{C}-E_{C}},\;\frac{\left(C_{R1\;}-C{\;}_{R2}\right)\left(1-b\right)-E_{R}-aP_{R}}{D_{R\;}-E_{R}})\;$is one of the stable points.

The stable point obtained from the replicator dynamics equation is not necessarily an ESS and needs to be further calculated according to the method proposed by Friedman [72]. Through the local stability analysis of the Jacobian matrix of the system, an ESS can be obtained. The Jacobian matrix *J* of this system is:

(23) $$J=\left[\begin{array}{cc} \frac{\partial F\left(x\right)}{\partial x}, & \frac{\partial F\left(x\right)}{\partial z}\\ \frac{\partial F\left(z\right)}{\partial x}, & \frac{\partial F\left(z\right)}{\partial z} \end{array}\right]=\left[\begin{array}{cc} a_{11} & a_{12}\\ a_{21} & a_{22} \end{array}\right]\;$$

Among them:

(24) $$a_{11}=\left(1-2x\right)\left[\left(1-b\right)\left(C_{R2}-C_{R1}\right)+E_{R}+aP_{R}+z\left(D_{R}-E_{R}\right)\right]$$

(25) $$a_{12}=x\left(1-x\right)\left(D_{R}-E_{R}\right)$$

(26) $$a_{21}=z\left(1-z\right)\left(D_{C}-E_{C}\right)$$

(27) $$a_{22}=\left(1-2z\right)[\left(I_{C}\left(1-\delta \right)-D_{C}+x\left(D_{C}-E_{C}\right)\right]\;$$

A stable point is judged as an EES if it satisfies the following conditions: (1) det (J) = a_11_a_22_ − a_12_a_21_ > 0; (2) Tr (J) = a_11_ + a_22_ < 0. Table 6 shows the values of a_11_, a_12_, a_21_, and a_22_ for each stable point. 

Note that det(J) represents the determinant, while Tr(J) represents the trace, which is the sum of the diagonal elements.

Let $\frac{\left(C_{R1}-C_{R2}\right)\left(1-b\right)-D_{R}}{P_{R}}=a_{1}$, $\frac{\left(C_{R1}-C_{R2}\right)\left(1-b\right)-E_{R}}{P_{R}}=a_{2}$, $1-\frac{D_{C}}{I_{C}}=\delta_{1}$, $1-\frac{E_{C}}{I_{C}}=\delta_{2}$, and ${0<a}_{1}{<a}_{2}{<1,\;\delta }_{1}{<\delta }_{2}<1$. Table 6 shows that Tr (J) is equal to 0 at the stable point of (x*, z*), which does not satisfy the condition that Tr (J) < 0 for the EES, so it is not an ESS of this system. Next, we discuss the stability of the remaining four equilibrium points—namely, (0,0), (0,1), (1,0), (1,1):

(1) When ${0<a<a}_{2}{,\delta }_{2}<\delta <1$ or $a_{1}{<a<a}_{2}{,\delta }_{2}<\delta <1$, ESS is (0, 0)
(2) When ${0<a<a}_{1}{,0<\delta <\delta }_{1}$, ESS is (0, 1)
(3) When $a_{2}{<a<1,\delta }_{2}<\delta <1$, ESS is (1, 0)
(4) When $a_{1}{<a<a}_{2}{,\delta }_{1}{<\delta <\delta }_{2}$, ESS is (0, 0) or (1, 1)
(5) When $a_{1}{<a<1,0<\delta <\delta }_{1}$ or $a_{1}{<a<a}_{2}{,0<\delta <\delta }_{1}$, ESS is (1, 1)

According to Table 6, the det (J) and Tr(J) values of each stable point can be calculated, and the stability under the above five conditions can be judged. The results are shown in Table 7, Table 8, Table 9, Table 10 and Table 11.

#### 4.1.2. Analysis of Evolutionary Stability in Case 4

**Figure 2:**
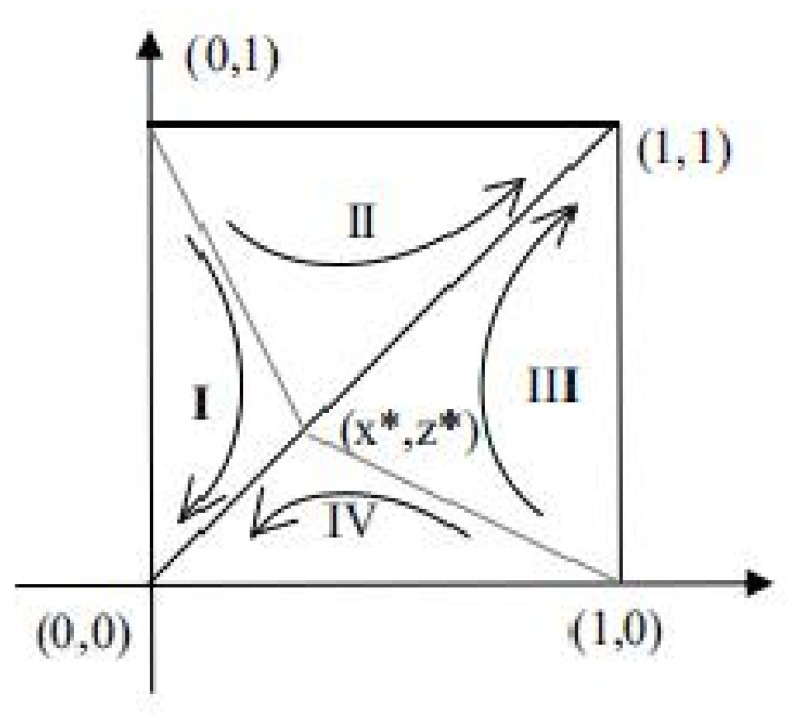
A phase diagram of the evolutionary game in case 4.

Case 4 is the most uncertain of the above five cases. When the evolutionary equilibrium point is in a stable state, the corresponding stable point may be either (0, 0) or (1, 1). This situation happens to be the most discussable situation, and it is also the closest to reality because both parties in the game want to maximize their profits and do not want their own interests to be damaged. Therefore, this manuscript chooses to discuss the influence of the parameters on the strategic evolution in case 4. Figure 2 shows the phase diagram of the evolutionary game in case 4. According to Figure 2, when $a_{1}<a<a_{2},\delta_{1}<\delta <\delta_{2}$, the ESS is (0, 0) or (1, 1). That is to say, no matter how the two parties initially make decisions, they will evolve towards either (strictly manufacture, active participation) or (tendency to formal manufacturing, passive participation) after continuous long-term games. Suppose that S1 is the sum of regions I and IV, while S2 is the sum of regions II and III. In the end, the strategy choice of both parties in the game is related to the relative size of S1 and S2. According to the analysis of the factors affecting the area change, the evolutionary direction of the system can be inferred.

(28) $$S1=\frac{1}{2}\left(x*+z*\right)=\frac{1}{2}\left[\frac{D_{C}-I_{C}\left(1-\delta \right)}{D_{C}-E_{C}}+\frac{\left(C_{R1}-C_{R2}\right)\left(1-b\right)-E_{R}-aP_{R}}{D_{R}-E_{R}}\right]$$

**Proposition** **1.** *Under the market mechanism, the probability of the two sides of the game choosing (tendency to formal manufacturing, passive participation) decreases as the government supervision rate a increases.*

**Proof.**  Because $\frac{\partial S1}{\partial a}=-\frac{P_{R}}{D_{R}-E_{R}}<0$, S1 is a monotonically decreasing function of a—that is, S1 will decrease with the increase in government supervision rate, and the probability of the system evolving to (tendency to formal manufacturing, passive participation) will decrease, with both sides of the game being more inclined to choose (strict manufacturing, active participation). □

**Proposition** **2.** *The stronger the leadership b of the CDW recycling unit and the smaller the additional costs incurred by strict manufacturing, the smaller the probability that both parties will choose (tendency to formal manufacturing, passive participation).*

**Proof.**  Because $\frac{\partial S1}{\partial b}=-\frac{C_{R1}{-C}_{R2}}{D_{R}{-E}_{R}}<0$, S1 is a monotonically decreasing function of b. As b increases, the area of S1 will decrease, and the probability of the system evolving to (1, 1) will increase. □

**Proposition** **3.** *The greater the consumer’s δ, the smaller the probability that both parties will choose (tendency to formal manufacturing, passive participation).*

**Proof.**  Because $\frac{\partial S1}{\partial \delta }=\frac{I_{C}}{D_{C}{-E}_{C}}>0$, S1 is a monotonically increasing function of δ. As δ increases, the area of S1 will increase, and the probability of the system evolving to (0, 0) will increase. □

The results are shown in Table 12. 

From the perspective of practical significance, the ideal goal is that the recycling unit chooses a strict manufacturing strategy, while the consumer chooses an active participation strategy—that is, strategy (1, 1). However, according to the analysis of the evolutionary game model in Section 4, since the internal performances of different companies and consumers are different, the final strategy choice in different situations is not necessarily (1, 1). Therefore, how best to make the recycling unit strictly manufacture remanufactured CDW products? How to make consumers participate more actively in the purchase of remanufactured CDW products? Here, factors such as consumers’ green preferences, government penalties, and corporate leadership need to be considered in order to achieve the stable development of the CDW recycling supply chain. We used MATLAB R2016b (MathWorks, Inc., Natick, Massachusetts, USA) software to simulate and analyze the ESS points of the evolutionary game system. Based on the literature review, the parameter settings for the benchmark situation (case 4) are shown in Table 13. While keeping the fixed parameters unchanged, the impact of other situations on the decision making of recycling units and consumers can be simulated by changing the variable parameters.

By consulting the literature, a construction waste production line with an hourly output of 100 tons can produce more than 0.7 tons of recycled aggregate per ton of construction waste [30,73]. According to the difference in aggregate strength, the price is roughly USD 4.164–12.305/ton, so the manufacturer of recycled aggregates needs to charge the builders USD 1.538–3.076 for every ton of construction waste processed. Hence, suppose that the average income of the production unit producing remanufactured CDW products is USD ~10.766/ton, so the value of $I_{R}$ is 70, while the basic cost $C_{C}$ paid by consumers is 55. The cost of construction waste treatment mainly includes three items: (1) equipment cost (USD ~5.306/ton); (2) site pretreatment cost (USD 1.077/ton for earthwork transportation, USD 0.200/ton for expenses); and (3) various insurance premiums (social insurance premiums are USD 0.038/ton, while taxes are USD 0.694/ton). These three costs add up to a total processing cost of USD ~7.690/ton. Therefore, if $C_{R2}$ is 50 and $C_{R1}$ is 70, the selling price of recycled aggregates is about 60% of the selling price of natural sand and gravel materials. In order to promote the production of remanufactured products by recycling units, the government assigns a value of 40 to $S_{R}$ (which is within the range of USD 3.076–21.533/ton of garbage disposal fee compensation provided by the government), and in the market, the government imposes a fine $P_{R}$ of 10 on recycling units that are inspected and found to tend towards formal manufacturing. Research on consumer premiums shows that consumers’ average premium for green agricultural products is ~20% [74]. Katrin [75] conducted research on willingness to pay for carbon-labeled products in six European countries; the results showed that the existence of carbon labels increases the probability of purchase, and consumers are willing to pay a price premium up to 20%, while local carbon-labeled products will also produce a higher “premium”. Therefore, suppose that the average highest price $I_{C}$ that actively participating consumers are willing to pay is USD 12.920.

Since the production unit can only continue to operate when its income is positive, and the loss of income generated by the consumer cannot be greater than the basic income of the recycling unit, then $0<D_{R}<I_{R}$. When consumers passively participate and the recycling unit strictly manufactures, and the consumer’s perceived loss $D_{C}$ will not exceed the basic cost $C_{C}$ paid by the consumer, then $0<D_{C}<C_{C}$. According to a survey by American Auto, each satisfied customer will affect 8 potential businesses, of which at least one is involved in the transaction; meanwhile, each unsatisfied customer will affect 25 people’s purchase intentions. Combined with the expert consultation method, the additional income $E_{R}$ generated by the production unit due to consumers is assigned a value of 2, and the loss $D_{R}$ is assigned a value of 8. The additional income $E_{C}$ generated by the consumer is 16 and the loss $D_{C}$ is assigned a value of 50.

Considering practical factors, the government cannot supervise all enterprises. At the same time, China’s government supervision system also has some drawbacks [76]. Therefore, the government’s supervision rate is 0.50 when other factors change, and the range of change is 0.2, 0.35, 0.5, 0.65, and 0.8 (where 0.2 is low efficiency and 0.8 is high efficiency). At present, there is no research on the difference in consumers’ willingness to pay δ for remanufactured products with different characteristics. In order to better study the influence of the difference in willingness to pay on the strategic choices of both parties, we assume that the value of the variable parameter δ is 0.6 when other parameters change, while the variation range is 0.4, 0.5, 0.6, 0.7, and 0.8. After more than 30 years of rapid economic development, China’s economy has formed a number of companies with strong capital, such as Alibaba, Wanda, and Lenovo; however, the leadership level of these companies is still far from that of outstanding companies in developed countries. Few Chinese companies have a large number of outstanding grassroots managers, as seen at companies such as GE and IBM [77]. At the same time, it can be seen from the existing literature that the quality of leadership of SMEs in China is generally not high [78], although not all of them are at a low level. Therefore, it is assumed that the variable parameter value b is 0.5 when other parameters change and that the range of change is 0, 0.25, 0.5, 0.75, and 1, which conforms to the classification of current leadership types: passive leadership, moderate leadership, and active leadership.

##### The Influence of Government Supervision Rate on the Evolutionary Results of Both Sides of the Game

**Figure 3:**
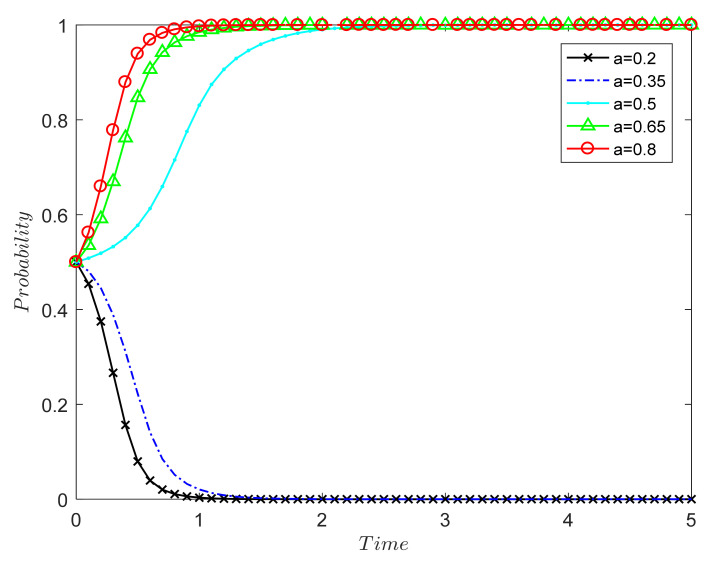
System evolution trajectory under different government supervision rates a.

The government supervision rate *a* is 0.2, 0.35, 0.5, 0.65, and 0.8, and the simulation results are shown in Figure 3. The numerical simulation results in Figure 3 verify Proposition 1: Under the market mechanism, the probability of the two parties in the game choosing (0, 0) decreases as the government supervision rate increases.

Between 0.2 and 0.35, the government’s regulatory power is too small, and it cannot encourage the two parties to evolve in the direction of (1, 1); in the end, the two parties choose (0, 0). When the supervision rate is 0.5 and above, the stability point of both parties in the game is (1, 1), and the greater the supervision rate, the faster the system reaches the stable point. Therefore, the government’s supervision and punishment can provide strong constraints for both parties, effectively avoiding the damage to consumers’ rights caused by the speculative behavior of recycling units and driving both parties to a stable strategy of (1, 1).

At present, most CDW remanufacturing units are in the formation and development stage, and their cost recovery period is relatively long. When the government supervision rate is not high, they are likely to choose to tend towards formal remanufacturing. However, most consumers are also on the sidelines of CDW remanufacturing, so the low supervision rate will reduce the willingness of both parties to choose (strictly, positively). Therefore, the government needs to strengthen supervision, establish a sound and dynamic CDW full-process supervision system and information disclosure system, and accurately record and manage the types, amounts, and whereabouts of construction waste in order to ensure that the whole process of construction waste—from dismantling, sorting, and transportation to processing and disposal—is standardized and orderly. Severe penalties must be imposed on the production units whose products are found to be unqualified in order to guide the sound development of the CDW recycling industry. Otherwise, production units may still choose speculative behavior that tends towards formal manufacturing, which will not only reduce the public’s recognition of remanufactured CDW products but also waste government resources and hinder the development of the CDW recycling supply chain.

##### The Influence of Recycling Unit Leadership on the Evolutionary Results of Both Parties in the Game

**Figure 4:**
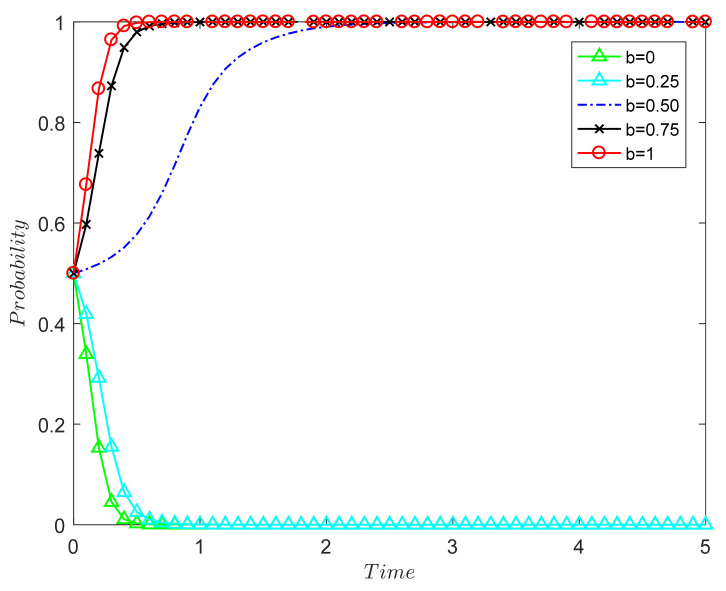
System evolution trajectory under different corporate leadership b.

In order to further explore the influence of corporate leadership on the outcome of the decision-making evolution of relevant units in the supply chain, Figure 4 simulates the evolution trajectory of the recycling unit when the leadership b is 0, 0.25, 0.5, 0.75, and 1.

The numerical simulation results in Figure 4 verify Proposition 2: The stronger the leadership b of the CDW recycling unit and the smaller the additional costs incurred by strict manufacturing, the smaller the probability that both parties will choose (tendency to formal manufacturing, passive participation). When b is small, the leadership of the recycling unit is too small, which cannot motivate both parties to evolve in the direction of (1, 1); in the end, both parties choose (0, 0). When the supervision rate is 0.5 or above, the stability point of both parties in the game is (1, 1), and the greater the leadership, the faster the system reaches the stable point. The stronger the leadership of the recycling unit, the more it will provide support for technical quality improvement, product marketing, etc., and further reduce the cost of strict manufacturing. The adoption of effective publicity strategies can also improve consumers’ environmental awareness and encourage consumers to actively purchase remanufactured CDW products. Therefore, the more mature the leadership of the recycling unit, the greater the profit obtained by both parties in the game, and the greater the probability that both parties will choose (1, 1). Thus, from a long-term point of view, production companies should start with themselves and consciously improve their own leadership level, rather than adopt a method that tends towards formal manufacturing in order to increase profits.

##### The Impact of the Consumer Payment Difference Coefficient on the Evolutionary Results of Both Parties in the Game

**Figure 5:**
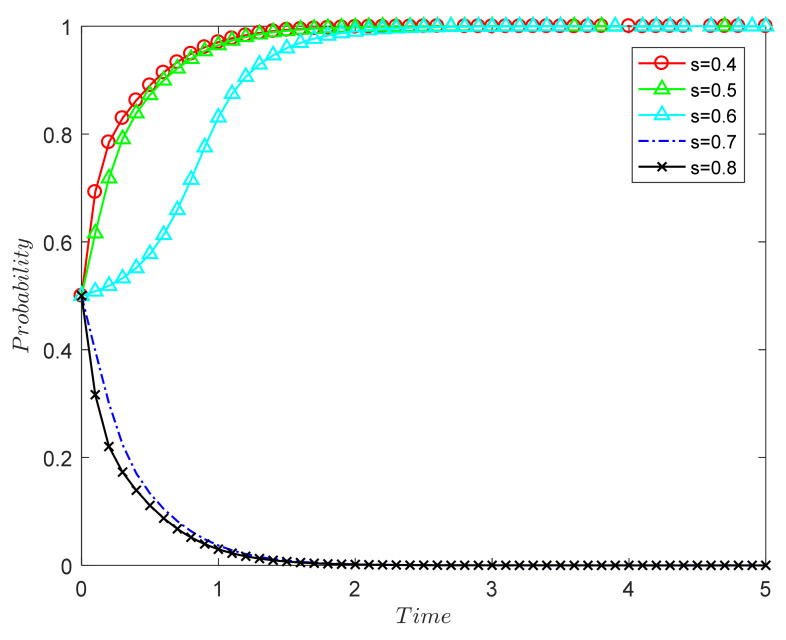
System evolution trajectory under different payment difference coefficients δ.

According to the above-mentioned stability analysis of the stable point, the ESS of the system will vary with different parameters. Based on the parameter settings, the influence of the parameters on the game balance can be simulated, as shown in Figure 5.

Note that the letter “s” in the figure represents the ratio of the highest price that passively participating consumers are willing to pay to that of actively participating consumers, i.e., δ.

When the other parameters remain unchanged and the δ value changes from 0.4 to 0.8, the ESS of the game model changes from (1, 1) to (0, 0), which is consistent with the analysis results pertaining to Proposition 3. It is not difficult to find that the larger the value of δ, the faster it converges to a stable state of passive participation. In other words, since the difference coefficient δ of the willingness to pay of passively participating consumers is larger than that of actively participating consumers, the maximum price difference between passively participating consumers and actively participating consumers is not large. In other words, the passively participating consumers at this time agree with the principle of remanufactured CDW products but, due to their own preferences, they may still choose to buy non-remanufactured products. Thus, in the event that the recycling unit adopts any kind of strategy, the benefits of consumers choosing passive participation may be greater than the benefits of active participation. Over time, the probability of bidirectional evolution to (0, 0) will increase.

For consumers, it is good to have environmental awareness, but they should not fall into the trap of green consumption. In real life, the government usually tends to adopt altruistic appeals to individuals for green consumption. Shi et al. [79] showed that the government can still adopt altruistic appeals in the stage of shaping green consumption attitudes. When consumers enter the actual purchase stage, companies can appropriately favor self-interested appeals, emphasizing the self-interested nature of remanufactured CDW products, or can create an egoistic green consumption situation in order to promote consumers to make their first consumption, guide consumers to gradually form good consumption demands, and avoid the “green consumption trap” that will follow. Consumers must distinguish right from wrong and prevent malicious use by enterprises.

### 4.2. In the Second Situation, the Game between Recycling Units and Third-Party Remanufacturers

#### 4.2.1. Calculation of Stable Points

According to Table 5, the expected payoffs of the CDW recycling unit for the strategies of *E* and *NE* are as follows:

(29) $$U_{E}=y\left[\left(1+k\right)\beta \pi_{p2}+M-C_{r}-∆C_{r}\right]+\left(1-y\right)\left[1+k\right)\beta \pi_{p1}+M-C_{r}-∆C_{r}$$

(30) $$U_{NE}=y\left(\beta \pi_{p2}+M-C_{r}\right)+\left(1-y\right)\beta \pi_{p1}+M-C_{r}$$

The expected payoffs of the CDW 3PR for the strategies of *E* and *NE* are as follows:

(31) $$U\prime_{E}=x\left[\left(1+k\right)\pi_{p2}\left(1-\beta \right)-M+\left(C_{p}+\Delta C_{p}\right)s\right]+\left(1-x\right)\left[\pi_{p2}\left(1-\beta \right)-M+\left(C_{p}+\Delta C_{p}\right)s\right]$$

(32) $$U\prime_{NE}=x\left[\left(1+k\right)\pi_{p1}\left(1-\beta \right)-M+C_{p}s\right]+\left(1-x\right)\left[\pi_{p1}\left(1-\beta \right)-M+C_{p}s\right]$$

Then, the replication dynamic equations can be established as follows:

(33) $$F\left(x\right)=\frac{dx}{dt}=x\left(1-x\right)\left(U_{E}-U_{NE}\right)=x\left(1-x\right)\left[yk\beta \left(\pi_{p2}-\pi_{p1}\right)+k\beta \pi_{p1}-\Delta C_{r}\right]$$

(34) $$F\left(y\right)=\frac{dy}{dt}=y\left(1-y\right)\left(U\prime_{E}-U\prime_{NE}\right)=y\left(1-y\right)\left[\left(\pi_{p2}-\pi_{p1}\right)\left(1-\beta \right)\left(xk+1\right)+\Delta C_{p}s\right]$$

According to the stability theory of first-order differential equations, if *dx*/*dt* = 0 and *dy*/*dt* = 0, the stable points of the system composed of Equations (38) and (39) can be obtained, i.e., (0, 0), (0, 1), (1, 0), and (1, 1). Naturally, (x*, y*) = ($\frac{-\Delta C_{p}s-\left(\pi_{p2}-\pi_{p1}\right)\left(1-\beta \right)}{\left(\pi_{p2}-\pi_{p1}\right)\left(1-\beta \right)k},\;\frac{\Delta C_{r}-k\beta \pi_{p1}}{k\beta \left(\pi_{p2}-\pi_{p1}\right)})\;$is one of the stable points. 

At this time, the Jacobian matrix is as follows:

(35) $$J=\left[\begin{array}{cc} \frac{\partial F\left(x\right)}{\partial x} & \frac{\partial F\left(x\right)}{\partial y}\\ \frac{\partial F\left(y\right)}{\partial x} & \frac{\partial F\left(y\right)}{\partial y} \end{array}\right]=\left[\begin{array}{cc} b_{11} & b_{12}\\ b_{21} & b_{22} \end{array}\right]$$

Among them:

(36) $$b_{11}=1-2x\left[yk\beta \left(\pi_{p2}-\pi_{p1}\right)+k\beta \pi_{p1}-\Delta C_{r}\right]$$

(37) $$b_{12}=x\left(1-x\right)\left[k\beta \left(\pi_{p2}-\pi_{p1}\right)\right]$$

(38) $$b_{21}=y\left(1-y\right)\left[k\left(\pi_{p2}-\pi_{p1}\right)\left(1-\beta \right)\right]$$

(39) $$b_{22}=\left(1-2y\right)\left[\left(\pi_{p2}-\pi_{p1}\right)\left(1-\beta \right)\left(xk+1\right)+\Delta C_{p}s\right]$$

A stable point is judged as an EES if it satisfies the following conditions: (1) det (J) = b_11_b_22_ − b_12_b_21_ > 0; (2) Tr (J) = b_11_ + b_22_ < 0. Table 14 shows the values of b_11_, b_12_, b_21_, and b_22_ for each stable point. 

Hypothesis $\frac{{(\pi }_{p1}{-\pi }_{p2})(1-\beta )}{{\Delta C}_{p}}{=s}_{1}$, $\frac{{(\pi }_{p1}{-\pi }_{p2})(1-\beta )(1+k)}{{\Delta C}_{p}}{=s}_{2}$, $\frac{{\Delta C}_{r}}{{\beta \pi }_{p1}}{=k}_{1}$, $\frac{{\Delta C}_{r}}{{\beta \pi }_{p2}}{=k}_{2}$. Table 14 shows that Tr (J) is equal to 0 at the stable point of (x*, y*), which does not satisfy the condition that Tr (J) < 0 for the EES, so it is not an ESS of this system. Next, we discuss the stability of the remaining four equilibrium points—namely, (0, 0), (0, 1), (1, 0), and (1, 1):

In summary, we can reach the following conclusions:

(1) When ${0<k<k}_{1}{,0<s<s}_{1}$, ESS is (0, 0);
(2) When $k_{1}{<k<k}_{2}{,s}_{2\;}{<s<1\mathrm{or}0<k<k}_{1}{,s}_{1}<s<1,\;$ESS is (0, 1);
(3) When $k_{2}{<k<1,0<s<s}_{2}{\;\mathrm{or}\;k}_{1\;}{<k<1,0<s<s}_{1}$,ESS is (1, 0);
(4) When $k_{1}{<k<k}_{2}{,s}_{1}{<s<s}_{2}$,ESS is (0, 1) or (1, 0);
(5) When $k_{2}{<k<1,s}_{2}<s<1$, ESS is (1, 1).

According to Table 16, the det(J) and Tr(J) values of each stable point can be calculated, and the stability results in the above five cases are shown in Table 15, Table 16, Table 17, Table 18 and Table 19.

In case (1), when $0<k<k_{1},0<s<s_{1}$, the ESS of this system is (0, 0). In this case, the profit increase coefficient brought by the high-quality efforts of the recycling unit for the 3PR is very low, and the government’s subsidy rate for the 3PR is also low. Regardless of whether the E strategy is selected alone or together, the benefits to both parties are relatively small, but both parties pay the cost for this. Therefore, both parties will choose the NE strategy in the end.

In case (2), when $k_{1}<k<k_{2},s_{2}<s<1$ or $0<k<k_{1},s_{1}<s<1$, the ESS of this system is (0, 1). In this case, the 3PR chooses the E strategy, and the recycling unit chooses the NE strategy. The recycling unit’s high-quality effort income increase coefficient increases, but the benefits from the 3PR unit cannot make up for the increased effort cost, so the recycling unit chooses the NE strategy. For 3PR units, the government’s subsidies for high-quality efforts are sufficient to balance the cost of high-quality efforts and the excess costs that need to be paid to the recycling unit, so the 3PR chooses the E strategy.

In case (3), when $k_{2}<k<1,0<s<s_{2}$ or $k_{1}<k<1,0<s<s_{1}$, the ESS of this system is (1, 0). In this case, after the recycling unit choosing high-quality efforts, the profit increase coefficient of the 3PR is further improved, and the recycling unit can obtain more benefits from the 3PR. For the 3PR, more funds need to be paid to the recovery unit, but the government subsidy rate does not increase significantly. The profit at this time is lower than that in the case of no effort, so the 3PR chooses the NE strategy.

In case (4), when $k_{1}<k<k_{2},s_{1}<s<s_{2}$, the ESS of this system is (1, 0) or (0, 1). In this case, both parties hope that the other party will choose high-quality efforts to generate more profits. When the recycling unit chooses high-quality efforts, the 3PR will choose low-quality efforts; when the 3PR chooses high-quality efforts, the recycling unit will choose low-quality efforts.

In case (5), when $k_{2}<k<1,s_{2}<s<1$, the ESS of this system is (1, 1). In this case, the profit increase coefficient of the recycling unit after choosing high-quality efforts is sufficiently large, as is the government’s subsidy rate to the 3PR. At this time, both the recycling unit and the 3PR both choose strategy E to obtain more profits than if the two chose strategy E from the other side and took NE by themselves. In this case, the recycling unit’s corporate management, production equipment, and production technology are all at an advanced level, so the profit increase coefficient after choosing high-quality efforts is sufficient to promote a substantial increase in the 3PR unit’s profit.

#### 4.2.2. Analysis of Evolutionary Stability Based on Parameter Changes (Case (4))

**Figure 6:**
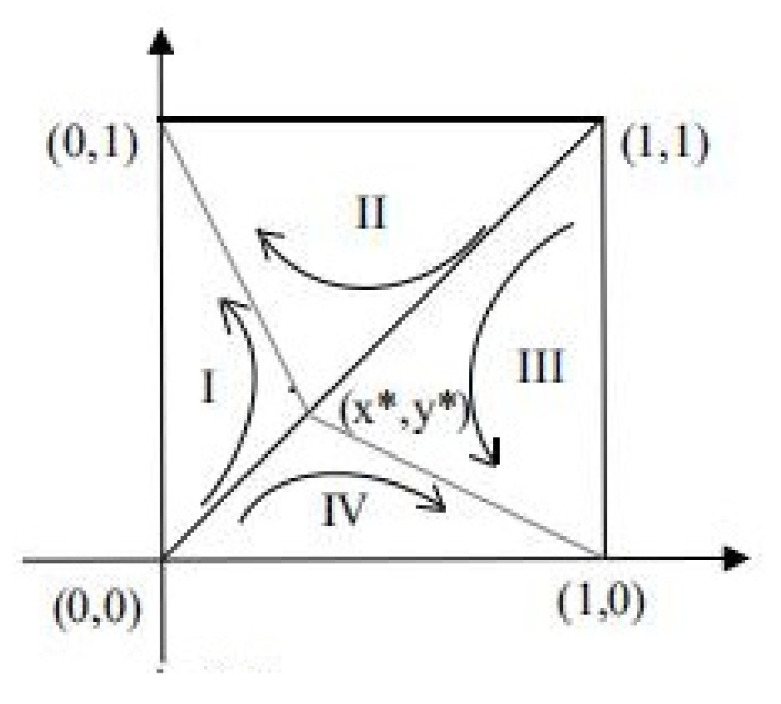
Phase diagram of the evolutionary game in case 4.

Since it is more common for both parties to participate in the game with medium performance, our study chooses case (4) for further analysis—namely, the influence of the parameters of $k_{1}<k<k_{2},s_{1}<s<s_{2}$ on the strategic evolution. Figure 6 shows the phase diagram of the evolutionary game in case (4).

Figure 6 shows that the square area is divided into four parts: I, II, III, and IV. Suppose that S3 is the sum of I and II, while S4 is the sum of III and IV. The ratio of S3 and S4 to the total strategy space depends on the initial value of each parameter in the model. In view of the fact that China’s CDW remanufacturing production supply chain cannot be of high quality, the initial situation in this field is (0, 0)—that is, recycling units and 3PRs are reluctant to choose high-quality efforts at the beginning. The probability that the recycling unit and the 3PR finally select the (0, 1) and (1, 0) strategies is determined by the ratio of S3 to S4 in the table.

(40) $$S3=\frac{1}{2}\left(1-y*\right)+\frac{1}{2}x*=\frac{1}{2}\left[1-\frac{\Delta C_{r}-k\beta \pi_{p1}}{k\beta \left(\pi_{p2}-\pi_{p1}\right)}+\frac{-\Delta C_{p}s-\left(\pi_{p2}-\pi_{p1}\right)\left(1-\beta \right)}{\left(\pi_{p2}-\pi_{p1}\right)\left(1-\beta \right)k}\right]$$

(41) $$S4=\frac{1}{2}y*+\frac{1}{2}\left(1-x*\right)=\frac{1}{2}\left[\frac{{\Delta C}_{r}-{k\beta \pi }_{p1}}{k\beta \left(\pi_{p2}-\pi_{p1}\right)}+1-\frac{-{\Delta C}_{p}s-\left(\pi_{p2}-\pi_{p1}\right)\left(1-\beta \right)}{\left(\pi_{p2}-\pi_{p1}\right)\left(1-\beta \right)k}\right]$$

**Proposition** **4.** *The recycling unit chooses low-quality efforts, and the probability of the 3PR choosing high-quality efforts decreases as the income increase coefficient increases.*

**Proof.**  $\frac{\partial S3}{\partial k}=\frac{1}{k^{2}}\left[\frac{\Delta C_{r}\left(1-\beta \right)+\Delta C_{p}s\beta +\left(\pi_{p2}-\pi_{p1}\right)\left(1-\beta \right)\beta }{\left(\pi_{p2}-\pi_{p1}\right)\left(1-\beta \right)\beta }\right]$, at this time, because $s_{1}<s<s_{2},\;s_{1}=\frac{\left(\pi_{p1}-\pi_{p2}\right)\left(1-\beta \right)}{\Delta C_{p}}$, so $\frac{\partial S3}{\partial k}<0$. S3 is a monotonically decreasing function of k. As k increases, the area of S3 will decrease, and the probability of the system evolving to the point (1, 0) will increase. □

**Proposition** **5.** *With the increase in government subsidies for the costs incurred by 3PRs, the 3PR choosing high-quality efforts will increase the probability that the recycling unit chooses low-quality efforts.*

**Proof.**  $\frac{\partial S3}{\partial s}=\frac{-\Delta C_{p}}{\left(\pi_{p2}-\pi_{p1}\right)\left(1-\beta \right)}>0$; S3 is a monotonically decreasing function of s. As s increases, the area of S3 will increase, and the probability of the system evolving to (0, 1) will increase. □

**Proposition** **6.** *The impact of the output profit share between the recycling unit and the 3PR on the final strategy choice of the game’s parties depends on the specific circumstances.*

**Proof.**  $\frac{\partial S3}{\partial \beta }=\frac{\Delta C_{r}{\left(1-\beta \right)}^{2}-\Delta C_{p}s\beta^{2}}{\beta^{2}k\left(\pi_{p2}-\pi_{p1}\right){\left(1-\beta \right)}^{2}}$; when $\Delta C_{r}{\left(1-\beta \right)}^{2}-\Delta C_{p}s\beta^{2}>0$ is satisfied, S3 is a decreasing function of β. As β increases, the probability of the system evolving to the point (0, 1) will decrease. Conversely, the probability of the system evolving to the point (1, 0) will increase. □

The results are shown in Table 20.

#### 4.2.3. Numerical Simulation and Discussion

Theoretically, the ideal ESS is that both the production unit and the 3PR choose high-quality efforts—that is, the strategy (1, 1). However, according to the analysis of the above evolutionary game model, due to their different performances, the best choice of strategy is not always (1, 1). Therefore, we used MATLAB to simulate and analyze the evolutionary game model and to intuitively discuss the influence of different parameters on the decision-making behavior of the recycling unit and the 3PR. Through the investigation of the literature, we conducted repeated discussions with relevant industry experts and scholars based on previous research along with the present situation and strived to generalize the conclusions. Our study adjusts the simulation data accordingly; the specific value does not represent the actual amount but rather the relative size difference between the various parameters. In case (4), the ranges of k and s are 0.194 < k < 0.333 and 0.533 < s < 0.693, respectively, and the initial values of k and s are thus determined. Table 21 shows the simulation parameter assignments.

##### The Effect of Government Cost Subsidy Rate on the Evolutionary Results of Both Parties in the Game

**Figure 7:**
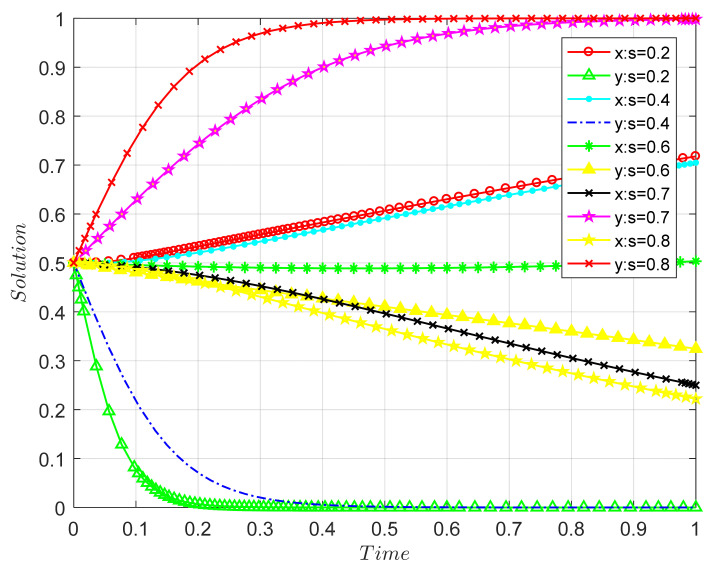
The influence of the government subsidy rate s on the dynamic evolution of the two parties in the game.

Combined with the specific cost subsidy policy, the cost subsidy coefficient s is set to 0.2, 0.4, 0.6, 0.7, and 0.8, and the simulation results are shown in Figure 7. The x and y in the figure refer to the respective probability of the recycling unit and the 3PR unit choosing high-quality efforts.

Figure 7 shows that when other parameters remain unchanged and the value of s changes from 0.2 to 0.8, the ESS of the model changes from (1, 0) to (0, 1), which is consistent with the analysis results of s shown in Table 20. The larger the value of s, the faster the 3PR will converge to a steady state of effort. When the cost-to-benefit ratio of the enterprise’s own effort cannot promote both parties evolving in the direction of (1,1), the government’s financial support will reduce the cost of the enterprise’s effort and encourage the enterprise to choose high-quality efforts. As a national key development industry, the CDW recycling industry is a pioneer that should have social responsibility. The development of the industry must pay more attention to social benefits, especially the ecological benefits that the state emphasizes. Therefore, government subsidies to enterprises can effectively reduce enterprise costs and encourage enterprises to work hard. At the same time, efforts should be made to cultivate consumers’ environmental awareness and to enable more consumers to become active participants instead of only buying remanufactured CDW products for subsidies and lower prices. Otherwise, once the government subsidies stop, remanufactured CDW products will no longer be purchased in large quantities, thus discouraging the enthusiasm of production units. Although, at the beginning of the period, the government may be “unpleasant”, only in this way can it truly achieve a win/win situation for all parties. At the same time, the government should increase its efforts to promote remanufactured CDW products so that more consumers can trust them. When consumers are willing to buy remanufactured CDW products, the government can appropriately lower the subsidies. In addition, it is necessary to comprehensively use multiple incentive policies to avoid a single subsidy policy.

##### The Influence of the Increase Coefficient of Recovered Unit Income on the Evolutionary Results of Both Parties

**Figure 8:**
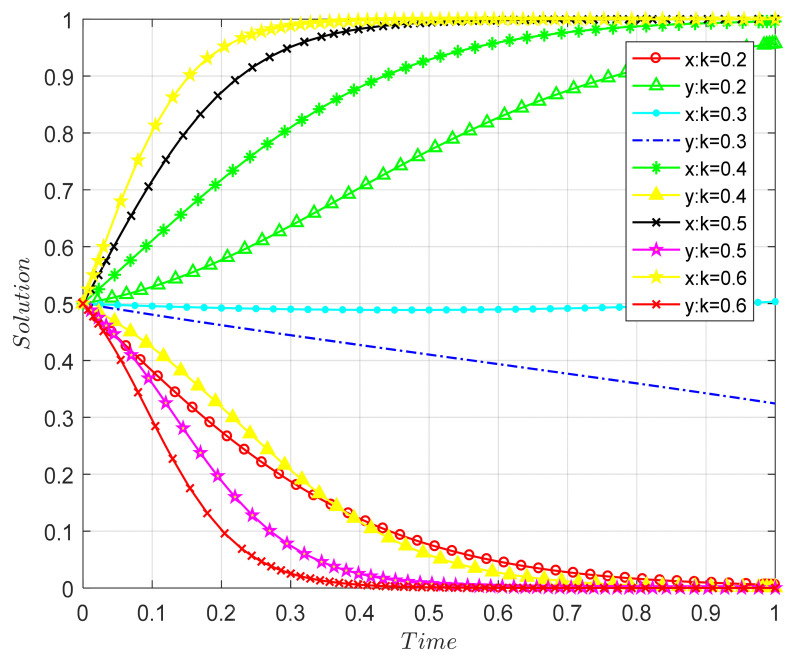
The effect of the profit increase coefficient k of the recycling unit on the evolution result of the two sides of the game.

As shown in Figure 8, when other parameters remain unchanged, as the value of k increases, the ESS will change from (0, 1) to (1, 0), which verifies Proposition 5. For the recycling unit, when k changes from 0.2 to 0.6, the recycling unit’s strategy will change from NE to E, and the larger the k, the faster the system converges to a steady state of effort. For the 3PR, as k increases, the 3PR will change from high-quality efforts to low-quality efforts. The larger the k, the faster the system converges to a stable state of effort—that is, the recycling unit makes full use of its own technological advantages to increase the profit of the 3PR in the CDW recycling supply chain through hard work, so as to obtain more rewards from the 3PR. However, the 3PR unit naturally hopes that the recycling unit can choose high-quality efforts so that it can enjoy the additional benefits in the process, and this part of the benefit is much greater than the additional subsidy from the government after the 3PR unit chooses a high-quality effort strategy. Therefore, this will prompt both parties to choose the (1, 0) strategy. Thus, recycling units and 3PR units should establish a reasonable cooperative incentive system and select appropriate coefficients. On the one hand, this can promote the recycling units to make efforts to recycle, and on the other hand, it will not make 3PR units feel that the cooperative incentive system is detrimental to their own interests.

## 5. Conclusions

Our study comprehensively considers government rewards and punishments, consumer green preference, leadership level, and other factors and establishes two game models of different types of recycling units with consumers and 3PRs, respectively. In addition, we conducted a theoretical analysis of the model, discussed the optimal strategies of the relevant units in different situations, and carried out further detailed analysis through calculation examples, reaching the following conclusions: (1) When the recycling unit has remanufacturing capabilities, corporate leadership and government supervision rate have a positive effect on its evolution to strict manufacturing, and a high supervision rate can effectively suppress the negative impact of speculative returns, driving the system’s evolution to (strict manufacturing, positive). The higher the supervision rate, the faster the system will converge. The consumer’s payment difference coefficient has a negative effect on the evolution of the recycling unit to strict manufacturing. The larger the payment difference coefficient, the faster it will converge to a stable state (tendency to formal remanufacturing, passive participation). (2) When the recycling unit does not have the ability to remanufacture, the government cost subsidy rate and the recycling unit’s effort profit coefficient have a positive effect on the recycling unit’s evolution in the direction of high-quality efforts, and the greater the coefficient of the recycling unit’s effort, the faster the system will converge. In the CDW construction waste recycling supply chain field, this study considers whether the recycling unit has only recently developed remanufacturing capabilities, and based on this, we conducted game analysis for two different situations. The previous literature often assumes that each recycling unit has remanufacturing capabilities. Thus, by considering the leadership factor and using simulations to verify the actual effect of the factor, this study also makes up for the lack of understanding of this aspect in the previous literature. Not only can this help to provide more scholars with new research ideas, but it also further enriches the relevant theoretical systems, such as the CDW remanufacturing and recycling supply chain, leadership, and evolutionary games. At the same time, it provides a theoretical basis for the recycling unit to make the best choice in the context of the new era. We believe that with the proposal of some advanced technologies [80,81] and the development of remanufacturing models, the harm inflicted on the construction industry will be greatly reduced.

However, this manuscript still has some limitations. First of all, we assume that the perceptual loss of passive participants is close to 0 when purchasing products that tend to be formally manufactured, but in real life, they will still experience a certain perceptual loss, albeit a very small one. Second, in order to simplify the model, this manuscript assumes that the relationship between leadership and cost is linear, but the actual situation may be more complicated. Moreover, the relationship between a recycling unit that does not have remanufacturing capabilities in the CDW recycling supply chain and a 3PR may involve cooperation and non-cooperation. Our study considers the game in the case of cooperation but not in the case of non-cooperation. 

Last but not least, how best to further analyze the degree of consumer response to quality issues? How best to accurately quantify the influence of leadership and consider the uncertainty of leadership in different periods? The continuous development of globalization will also extend the design of closed-loop supply chain networks from regional to global scope; thus, more complex, realistic conditions and assumptions need to be considered. Furthermore, the contents of this paper mainly focus on the Chinese construction industry; we hope that other researchers can carry out a further study based on the backgrounds of various other countries. Accordingly, we recommend that future research should be aimed at validating the theoretical numerical model with real cases and construction sites. 

## Figures and Tables

**Table 1:** The summary of the assumptions in the evolutionary game.

Assumption	Content
Assumption 1	The three parties involved are all economic entities with bounded rationality.
Assumption 2	The government’s subsidy to the recycling unit comes before the recycling process, while the government’s subsidy to consumers comes after the consumers’ purchase of remanufactured CDW products.
Assumption 3	In the first situation, the recycling unit has two strategies: (1) strictly producing remanufactured products (AP); or (2) tendency to formally produce manufactured products (F).
Assumption 4	In the first situation, consumers have two strategies: (1) active participation (A); or (2) passive participation (N).
Assumption 5	The actual cost of producing remanufactured CDW products is as follows: $C_{r1,2}=(1\;-{\;b)C}_{R1,2}$, where b is the leadership of the management (0<b<1).
Assumption 6	The highest price that consumers are willing to pay for actively participating is $I_{C}$, while the highest price that passively participating consumers are willing to pay is $\delta I_{C}$, $\delta \in \left(0,1\right)$.
Assumption 7	Consumers will experience perceived loss $D_{C}$ when consumers choose strategy A and recycling units choose strategy F. When they choose strategy N and recycling units choose strategy AP, they can generate perceived benefits $E_{C}$. In addition, they do not produce perceived gains or losses in the other two possible situations.
Assumption 8	In the second situation, the strategies of both the recycling unit and the 3PR are either (1) quality effort(E); or (2) not quality effort (EN).
Assumption 9	There is a linear incentive contract between 3PRs and recyclers, as follows: $S\left(\pi_{p}\right)$ = M + $\beta \pi_{p}$.
Assumption 10	The government subsidy is an ex post reward; the subsidy rate is r and the subsidy fee is C∗r.

**Table 2:** Related parameters and description of the main body of the game in the first situation.

Game Subject	Parameter	Parameter Description
Recycling unit (R)	x	Probability that the recycling unit chooses “strict manufacturing” in the first situation.
	(1−x)	Probability that the recycling unit chooses “tendency to manufacture” in the first situation.
	$I_{R}$	The basic income from producing remanufactured CDW products.
	$S_{R}$	Government subsidies obtained after producing remanufactured CDW products.
	$C_{R1}$	The cost of strictly producing remanufactured CDW products.
	$E_{R}$	Additional benefits such as economic and social benefits brought by consumers who passively participate in the purchase and recognition of remanufactured CDW products.
	$D_{R}$	The loss caused by the tendency of formally remanufactured CDW products to disappoint the active consumers, including tangible and intangible losses; tangible means the loss of a group of trusted customers, while intangible means the loss of corporate reputation.
	$P_{R}$	The fines incurred by recycling units that tend towards formal manufacturing after being discovered by the government.
	a	Probability of tendency towards formal manufacturing being discovered by the government. (0<a<1)
	b	Management leadership. (0<b<1)
Consumer (C)	z	Probability that the consumer is “active”.
	(1−z)	Probability that the consumer is “negative”.
	$I_{C}$	The highest price that consumers who actively participate are willing to pay.
	$C_{C}$	Basic cost paid by consumers
	δ	The ratio of the highest price that passively participating consumers are willing to pay to that of actively participating consumers. (0<δ<1)
	$D_{C}$	The consumer’s perceived loss when actively participating consumers and the recycling unit choose “tendency to manufacture”.
	$E_{C}$	The perceived benefits generated by consumers when passively participating consumers and the recycling unit choose “strict manufacturing”.
	$S_{C}$	Government subsidies to consumers who purchase remanufactured products.

**Table 3:** Game profit matrix between recycling units and consumers in the first situation.

Both Sides of the Game	Consumer (C)	
	Positive (z)	Negative (1−z)
Recycling unit (R)	Strict manufacturing(x) $\left(u_{r1},u_{c1}\right)$	$\left(u_{r2},u_{c2}\right)$
	Tend towards formal manufacturing (1 −x) $\;\left(u_{r3},u_{c3}\right)$	$\left(u_{r4},u_{c4}\right)$

**Table 4:** Related parameters and description of the main body of the game in the second situation.

Game Subject	Parameter	Parameter Description
Recycling unit (R)	x	Probability of “high-quality effort” of the recycling unit.
	(1−x)	Probability of “low-quality effort” of the recycling unit.
	$C_{r}$	The basic cost of recycling CDW.
	${\Delta C}_{r}$	The cost of recycling unit’s effort.
	β	The share of the output profit shared by the 3PR using CDW remanufacturing under the cooperative incentive mechanism. (0<β<1)
Third-party remanufacturers (3PRs)	y	Probability of the 3PR choosing “high-quality effort”.
	(1−y)	Probability of the 3PR choosing “low-quality effort”.
	$\pi_{p1}$	The profit that the 3PR produces using CDW under the condition that recycling units and the 3PR choose “low-quality effort”. ($\pi_{p1}>0)$
	$\pi_{p2}$	The profit that the 3PR produces using CDW in the case that recycling units choose “low-quality effort” and the 3PR chooses “high-quality effort”. ($\pi_{p2}>0$)
	$C_{p}$	The cost of basic remanufacturing to the 3PR.
	${\Delta C}_{p}$	The cost of the 3PR choosing “high-quality effort”.
Others	k	The income increase coefficient when recycling units choose “high-quality effort”. (0<k<1)
	s	The government subsidy rate to the 3PR. (0<s<1)

**Table 5:** Game profit matrix between the recycling unit and the 3PR in the second situation.

Both Sides of the Game	3PR (P)	
	High-Quality Effort (y)	Low-Quality Effort (1−y)
Recycling unit (R)	High-quality Effort (x) $\left(u_{r1},u_{p1}\right)$	$\left(u_{r2},u_{p2}\right)$
	Low-quality Effort (1−x)$\;(u_{r3},u_{p3})$	$\left(u_{r4},u_{p4}\right)$

**Table 6:** The value of each partial equilibrium point.

Equilibrium Point	a11	a12	a21	a22
(0, 0)	${(1-b)(C}_{R2}-C_{R1}{)+E}_{R\;}{+\mathrm{aP}}_{R}$	0	0	$I_{C}(1-\delta )-D_{C}$
(0, 1)	${(1-b)(C}_{R2\;}-C_{R1}{)+\mathrm{aP}}_{R}{+D}_{R}$	0	0	$-{[I}_{C}(1-\delta )-D_{C}]$
(1, 0)	$-{[(1-b)(C}_{R2}-C_{R1}{)+E}_{R\;}{+\mathrm{aP}}_{R}]$	0	0	$I_{C}(1-\delta )-E_{C}$
(1, 1)	$-{[(1-b)(C}_{R2}-C_{R1}{)+\mathrm{aP}}_{R}{+D}_{R}]$	0	0	$-{[I}_{C}(1-\delta )-E_{C}]$
(x*, z*)	0	-	-	0

Note: Since the values of a12 and a21 in (x*, z*) have nothing to do with the analysis, they are not calculated.

**Table 7:** Stability analysis of the points in case (1).

Point	${0<a<a}_{2}{,\delta }_{2}<\delta <1$			$a_{1}{<a<a}_{2}{,\delta }_{2}<\delta <1$		
	Det(J)	Tr(J)	Stability	Det(J)	Tr(J)	Stability
(0, 0)	+	-	ESS	+	-	ESS
(0, 1)	−	?	Saddle point	+	+	Unstable point
	+	+	Unstable point			
(1, 0)	−	?	Saddle point	+	+	Unstable point
				−	?	Saddle point
(1, 1)	−	?	Saddle point	−	?	Saddle point
	+	+	Unstable point			

Note that “+” means that the calculation result is greater than 0, “−” means that it is less than 0, and “?” means that it is uncertain. ESS: evolutionary stability strategy.

**Table 8:** Stability analysis of the points in case (2).

Point	${0<a<a}_{1}{,\;0<\delta <\delta }_{1}$		
	Det(J)	Tr(J)	Stability
(0, 0)	−	?	Saddle point
(0, 1)	+	−	ESS
(1, 0)	+	+	Unstable point
(1, 1)	−	?	Saddle point

**Table 9:** Stability analysis of the points in case (3).

Point	$a_{2}{<a<1,\delta }_{2}<\delta <1$		
	Det(J)	Tr(J)	Stability
(0, 0)	−	?	Saddle point
(0, 1)	+	+	Unstable point
(1, 0)	+	−	ESS
(1, 1)	−	?	Saddle point

**Table 10:** Stability analysis of the points in case (4).

Point	$a_{1}{<a<a}_{2}{,\delta }_{1}{<\delta <\delta }_{2}$		
	Det(J)	Tr(J)	Stability
(0,0)	+	−	ESS
(0,1)	+	+	Unstable point
(1,0)	+	+	Unstable point
(1,1)	+	−	ESS

**Table 11:** Stability analysis of the points in case (5).

Point	$a_{1}{<a<1,0<\delta <\delta }_{1}$			$a_{1}{<a<a}_{2}{,0<\delta <\delta }_{1}$		
	Det (J)	Tr (J)	Stability	Det(J)	Tr (J)	Stability
(0, 0)	−	?	Saddle point	−	?	Saddle point
	+	+	Unstable point			
(0, 1)	−	?	Saddle point	−	?	Saddle point
(1, 0)	−	?	Saddle point	+	+	Unstable point
(1, 1)	+	−	ESS	+	−	ESS

**Table 12:** Correlation analysis of the parameters in the first system.

Parameter	a	b	δ
	↑	↑	↑
**S1**	↓	↓	↑

**Table 13:** Correlation analysis results of relevant parameters in the model.

The Form of the Parameter	Parameter Setting	Parameter Value
Fixed parameter	$I_{R}$	70
	$S_{R}$	40
	$C_{R1}$	70
	$C_{R2}$	50
	$E_{R}$	2
	$D_{R}$	8
	$P_{R}$	10
	$I_{C}$	84
	$C_{C}$	55
	$D_{C}$	50
	$E_{C}$	16
	$S_{C}$	20
Variable parameter	a	0.5
	δ	0.6
	b	0.5

**Table 14:** The value of each partial equilibrium point.

Equilibrium Point	b11	b12	b21	b22
(0, 0)	${k\beta \pi }_{p1}-{\Delta C}_{r}$	0	0	${(\pi }_{p2}-\pi_{p1})(1-{\beta )+\Delta C}_{p}s$
(0, 1)	${k\beta \pi }_{p2}-{\Delta C}_{r}$	0	0	$-{[(\pi }_{p2}-\pi_{p1})(1-{\beta )+\Delta C}_{p}s]$
(1, 0)	$-{(k\beta \pi }_{p1}-{\Delta C}_{r})$	0	0	${(\pi }_{p2}-\pi_{p1})(1-{\beta )(k+1)+\Delta C}_{p}s$
(1, 1)	$-{(k\beta \pi }_{p2}-{\Delta C}_{r})$	0	0	$-{[(\pi }_{p2}-\pi_{p1})(1-{\beta )(k+1)+\Delta C}_{p}s]$
(x*, y*)	0	−	−	0

**Table 15:** Stability analysis of the points in case (1).

Point	${0<k<k}_{1}{,0<s<s}_{1}$		
	Det (J)	Tr (J)	Stability
(0, 0)	+	−	ESS
(0, 1)	−	?	Saddle point
(1, 0)	−	?	Saddle point
(1, 1)	+	+	Unstable point

**Table 16:** Stability analysis of the points in case (2).

Point	$k_{1}{<k<k}_{2}{,\;s}_{2}<s<1$			${0<k<k}_{1}{,\;s}_{1}<s<1$		
	Det (J)	Tr (J)	Stability	Det (J)	Tr (J)	Stability
(0, 0)	+	+	Unstable point	−	?	Saddle point
(0, 1)	+	−	ESS	+	−	ESS
(1, 0)	−	?	Saddle point	+	+	Unstable point
				−	?	Saddle point
(1, 1)	−	?	Saddle point	+	+	Unstable point
				−	?	Saddle point

**Table 17:** Stability analysis of the points in case (3).

Point	$k_{2}{<k<1,\;0<s<s}_{2}$			$k_{1}{<k<1,\;0<s<s}_{1}$		
	Det (J)	Tr (J)	Stability	Det (J)	Tr (J)	Stability
(0, 0)	−	?	Saddle point	−	?	Saddle point
	+	+	Unstable point			
(0, 1)	+	+	Unstable point	−	?	Saddle point
	−	?	Saddle point	+	+	Unstable point
(1, 0)	−	+	ESS	−	+	ESS
(1, 1)	−	?	Saddle point	+	+	Unstable point
				−	?	Saddle point

**Table 18:** Stability analysis of the points in case (4).

Point	$k_{1}<k<k_{2},s_{1}<s<s_{2}$		
	Det (J)	Tr (J)	Stability
(0, 0)	+	+	Unstable point
(0, 1)	−	+	ESS
(1, 0)	−	+	ESS
(1, 1)	+	+	Unstable point

**Table 19:** Stability analysis of the points in case (5).

Point	$k_{2}{<k<1,\;s}_{2}<s<1$		
	Det (J)	Tr (J)	Stability
(0, 0)	+	−	Unstable point
(0, 1)	−	?	Saddle point
(1, 0)	−	?	Saddle point
(1, 1)	+	−	ESS

**Table 20:** Correlation analysis of the parameters in the second system.

**Parameter**	**k**	**s**	**β**
	↑	↑	↑
**S3**	↓	↑	U ^1^

^1^ indicates that the correlation of the parameter is uncertain.

**Table 21:** Simulation parameter assignment.

Parameter	$\Delta C_{r}$	$\Delta C_{p}$	$\pi_{p1}$	$\pi_{p2}$	k	β	s
Value	200	600	2500	2000	0.3	0.2	0.6

## Data Availability

Not applicable.
